# Outpatient COVID-19 convalescent plasma recipient antibody thresholds correlated to reduced hospitalizations within a randomized trial

**DOI:** 10.1172/jci.insight.178460

**Published:** 2024-03-14

**Authors:** Han-Sol Park, Anna Yin, Caelan Barranta, John S. Lee, Christopher A. Caputo, Jaiprasath Sachithanandham, Maggie Li, Steve Yoon, Ioannis Sitaras, Anne Jedlicka, Yolanda Eby, Malathi Ram, Reinaldo E. Fernandez, Owen R. Baker, Aarthi G. Shenoy, Giselle S. Mosnaim, Yuriko Fukuta, Bela Patel, Sonya L. Heath, Adam C. Levine, Barry R. Meisenberg, Emily S. Spivak, Shweta Anjan, Moises A. Huaman, Janis E. Blair, Judith S. Currier, James H. Paxton, Jonathan M. Gerber, Joann R. Petrini, Patrick B. Broderick, William Rausch, Marie Elena Cordisco, Jean Hammel, Benjamin Greenblatt, Valerie C. Cluzet, Daniel Cruser, Kevin Oei, Matthew Abinante, Laura L. Hammitt, Catherine G. Sutcliffe, Donald N. Forthal, Martin S. Zand, Edward R. Cachay, Jay S. Raval, Seble G. Kassaye, Christi E. Marshall, Anusha Yarava, Karen Lane, Nichol A. McBee, Amy L. Gawad, Nicky Karlen, Atika Singh, Daniel E. Ford, Douglas A. Jabs, Lawrence J. Appel, David M. Shade, Bryan Lau, Stephan Ehrhardt, Sheriza N. Baksh, Janna R. Shapiro, Jiangda Ou, Yu Bin Na, Maria D. Knoll, Elysse Ornelas-Gatdula, Netzahualcoyotl Arroyo-Curras, Thomas J. Gniadek, Patrizio Caturegli, Jinke Wu, Nelson Ndahiro, Michael J. Betenbaugh, Alyssa Ziman, Daniel F. Hanley, Arturo Casadevall, Shmuel Shoham, Evan M. Bloch, Kelly A. Gebo, Aaron A.R. Tobian, Oliver Laeyendecker, Andrew Pekosz, Sabra L. Klein, David J. Sullivan

**Affiliations:** 1 W. Harry Feinstone Department of Molecular Microbiology and Immunology, Johns Hopkins Bloomberg School of Public Health, Baltimore, Maryland, USA.; 2 Department of Pathology, Johns Hopkins University School of Medicine, Baltimore, Maryland, USA.; 3 Department of International Health, Johns Hopkins Bloomberg School of Public Health, Baltimore, Maryland, USA.; 4 Department of Medicine, Division of Infectious Diseases, Johns Hopkins University School of Medicine, Baltimore, Maryland, USA.; 5 Department of Medicine, Division of Hematology and Oncology, MedStar Washington Hospital Center, Washington DC, USA.; 6 Division of Allergy and Immunology, Department of Medicine, NorthShore University Health System, Evanston, Illinois, USA.; 7 Department of Medicine, Section of Infectious Diseases, Baylor College of Medicine, Houston, Texas, USA.; 8 Department of Medicine, Division of Pulmonary and Critical Care Medicine, University of Texas Health Science Center, Houston, Texas, USA.; 9 Department of Medicine, Division of Infectious Diseases, University of Alabama at Birmingham, Birmingham, Alabama, USA.; 10 Department of Emergency Medicine, Rhode Island Hospital, Brown University, Providence, Rhode Island, USA.; 11 Luminis Health, Annapolis, Maryland, USA.; 12 Department of Medicine, Division of Infectious Diseases, University of Utah School of Medicine, Salt Lake City, Utah, USA.; 13 Department of Medicine, Division of Infectious Diseases, University of Miami Miller School of Medicine, Miami, Florida, USA.; 14 Department of Medicine, Division of Infectious Diseases, University of Cincinnati, Cincinnati, Ohio, USA.; 15 Department of Medicine, Division of Infectious Diseases, Mayo Clinic Hospital, Phoenix, Arizona, USA.; 16 Department of Medicine, Division of Infectious Diseases, UCLA, Los Angeles, California, USA.; 17 Department of Emergency Medicine, Wayne State University School of Medicine, Detroit, Michigan, USA.; 18 Department of Medicine, Division of Hematology and Oncology, University of Massachusetts Chan Medical School, Worcester, Massachusetts, USA.; 19 Nuvance Health, Danbury, Connecticut, USA.; 20 Nuvance Health Danbury Hospital, Danbury, Connecticut, USA.; 21 Nuvance Health Norwalk Hospital, Norwalk, Connecticut, USA.; 22 Nuvance Health Vassar Brothers Medical Center, Poughkeepsie, New York, USA.; 23 Ascada Research, Fullerton, California, USA.; 24 Department of Medicine, Division of Infectious Diseases, University of California, Irvine, California, USA.; 25 Department of Medicine, University of Rochester Medical Center, Rochester, New York, USA.; 26 Department of Medicine, Division of Infectious Diseases, UCSD, San Diego, California, USA.; 27 Department of Pathology, University of New Mexico School of Medicine, Albuquerque, New Mexico, USA.; 28 Department of Medicine, Division of Infectious Diseases, Georgetown University Medical Center, Washington DC, USA.; 29 Department of Neurology, Brain Injury Outcomes,; 30 Institute for Clinical and Translational Research, and; 31 Department of Ophthalmology, Johns Hopkins University School of Medicine, Baltimore, Maryland, USA.; 32 Department of Epidemiology, Johns Hopkins Bloomberg School of Public Health, Baltimore, Maryland, USA.; 33 Welch Center for Prevention, Epidemiology and Clinical Research, Johns Hopkins University School of Medicine, Baltimore, Maryland, USA.; 34 Chemistry-Biology Interface Program, Zanvyl Krieger School of Arts & Sciences, Johns Hopkins University, Baltimore, Maryland, USA.; 35 Department of Pharmacology and Molecular Sciences, Johns Hopkins University School of Medicine, Baltimore, Maryland, USA.; 36 Department of Pathology and Laboratory Medicine, Northshore University Health System, Evanston, Illinois, USA.; 37 Advanced Mammalian Biomanufacturing Innovation Center, Department of Chemical and Biomolecular Engineering, Johns Hopkins University, Baltimore, Maryland, USA.; 38 Department of Pathology and Laboratory Medicine, Wing-Kwai and Alice Lee-Tsing Chung Transfusion Service, David Geffen School of Medicine, UCLA, Los Angeles, California, USA.; 39 Division of Intramural Research, National Institute of Allergy and Infectious Diseases (NIAID), Baltimore, Maryland, USA.

**Keywords:** COVID-19, Immunoglobulins, Immunotherapy

## Abstract

**BACKGROUND:**

COVID-19 convalescent plasma (CCP) virus-specific antibody levels that translate into recipient posttransfusion antibody levels sufficient to prevent disease progression are not defined.

**METHODS:**

This secondary analysis correlated donor and recipient antibody levels to hospitalization risk among unvaccinated, seronegative CCP recipients within the outpatient, double-blind, randomized clinical trial that compared CCP to control plasma. The majority of COVID-19 CCP arm hospitalizations (15/17, 88%) occurred in this unvaccinated, seronegative subgroup. A functional cutoff to delineate recipient high versus low posttransfusion antibody levels was established by 2 methods: (i) analyzing virus neutralization–equivalent anti–Spike receptor-binding domain immunoglobulin G (anti-S-RBD IgG) responses in donors or (ii) receiver operating characteristic (ROC) curve analysis.

**RESULTS:**

SARS-CoV-2 anti–S-RBD IgG antibody was volume diluted 21.3-fold into posttransfusion seronegative recipients from matched donor units. Virus-specific antibody delivered was approximately 1.2 mg. The high-antibody recipients transfused early (symptom onset within 5 days) had no hospitalizations. A CCP-recipient analysis for antibody thresholds correlated to reduced hospitalizations found a statistical significant association between early transfusion and high antibodies versus all other CCP recipients (or control plasma), with antibody cutoffs established by both methods-donor-based virus neutralization cutoffs in posttransfusion recipients (0/85 [0%] versus 15/276 [5.6%]; *P* = 0.03) or ROC-based cutoff (0/94 [0%] versus 15/267 [5.4%]; *P* = 0.01).

**CONCLUSION:**

In unvaccinated, seronegative CCP recipients, early transfusion of plasma units in the upper 30% of study donors’ antibody levels reduced outpatient hospitalizations. High antibody level plasma units, given early, should be reserved for therapeutic use.

**TRIAL REGISTRATION:**

ClinicalTrials.gov NCT04373460.

**FUNDING:**

Department of Defense (W911QY2090012); Defense Health Agency; Bloomberg Philanthropies; the State of Maryland; NIH (3R01AI152078-01S1, U24TR001609-S3, 1K23HL151826NIH); the Mental Wellness Foundation; the Moriah Fund; Octapharma; the Healthnetwork Foundation; the Shear Family Foundation; the NorthShore Research Institute; and the Rice Foundation.

## Abstract 2

Therapeutic COVID-19 convalescent plasma donor units should be restricted to higher antibody levels in the top 30% with transfusion early after symptom onset.

## Graphical abstract

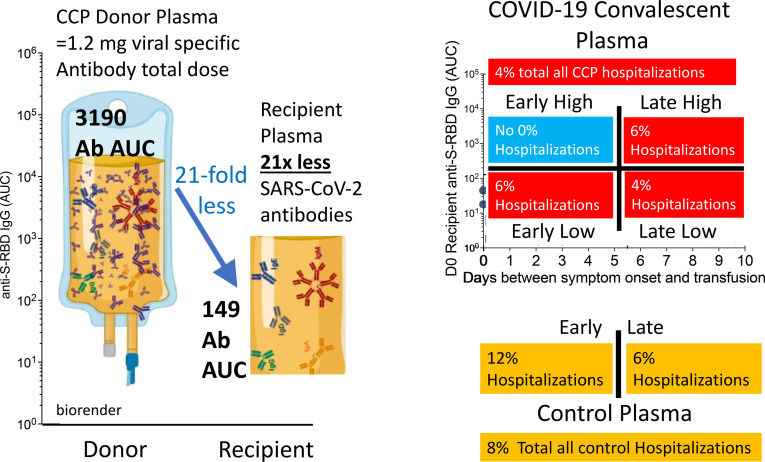

## Introduction

The SARS-CoV-2–specific antibody levels necessary to prevent infection or reduce hospitalization from mild outpatient COVID-19 or reduce deaths in those already hospitalized are likely to be different. For hospitalized patients, effective COVID-19 convalescent plasma (CCP) antibody levels have been estimated from registries (1, 2), but comparable information is not available for outpatient usage. The high interlaboratory variability with diverse SARS-CoV-2 serologic assays for binding or virus neutralizing antibody (nAb) levels creates further challenges (3, 4). Dilutional live or pseudovirus neutralization measures from 27 separate pre-Alpha convalescent plasma collections varied in geometric means (GMs) for 50% inhibition from 19 to 4,344, with a mean of 311 (5). Separating protective antibody metrics in vaccinated people or COVID-19 convalescent plasma donors that are still therapeutic after dilution into recipients further adds to complexity. For example, influenza vaccinees in the 1970s with dilutional virus hemagglutination inhibition titer of 1:40 or higher prevented infection (6, 7), such that the World Health Organization set the threshold of protection at 1:40 (8). Infants with respiratory syncytial virus in 2 separate studies with nAb titers over 1:256 are protected from hospitalizations (9, 10). However, therapeutic convalescent plasma would need to have 10–20 times the protective neutralization titer after a small plasma volume is diluted into a seronegative recipient.

CCP has proven effective by randomized controlled trials (RCTs) in 3 phases of COVID-19: outpatients (5, 11), inpatients (12, 13), and those within 48 hours of invasive mechanical ventilation (14). Many RCTs were stopped prematurely, transfused low to no SARS-CoV-2 specific antibody, or were given too late in disease progression to have antibody antiviral action change the disease course (15). Early CCP transfusion with high levels of antibodies is effective.

We previously reported that outpatient transfusion randomized to CCP or control plasma in 1,181 participants with pre-Delta CCP reduced the risk of hospitalization by 54% (5). A prespecified analysis from the parent outpatient CCP RCT aimed to compare antibody levels in donor-recipient pairs to explore the association between antibody levels and prevention of hospitalizations in recipients. With 88% of posttransfusion COVID-19 hospitalizations (15 of 17 total) occurring among unvaccinated, seronegative outpatient recipients, we analyzed hospitalization risk among this group by comparing CCP recipients stratified by early or late treatment (i.e., ≤5 versus >5 days from symptom onset) with antibody levels to demarcate pre-Delta CCP for pre-Omicron recipient thresholds for efficacy in reducing mild CoVID-19 hospitalizations.

## Results

### Trial population.

**Figure 1:**
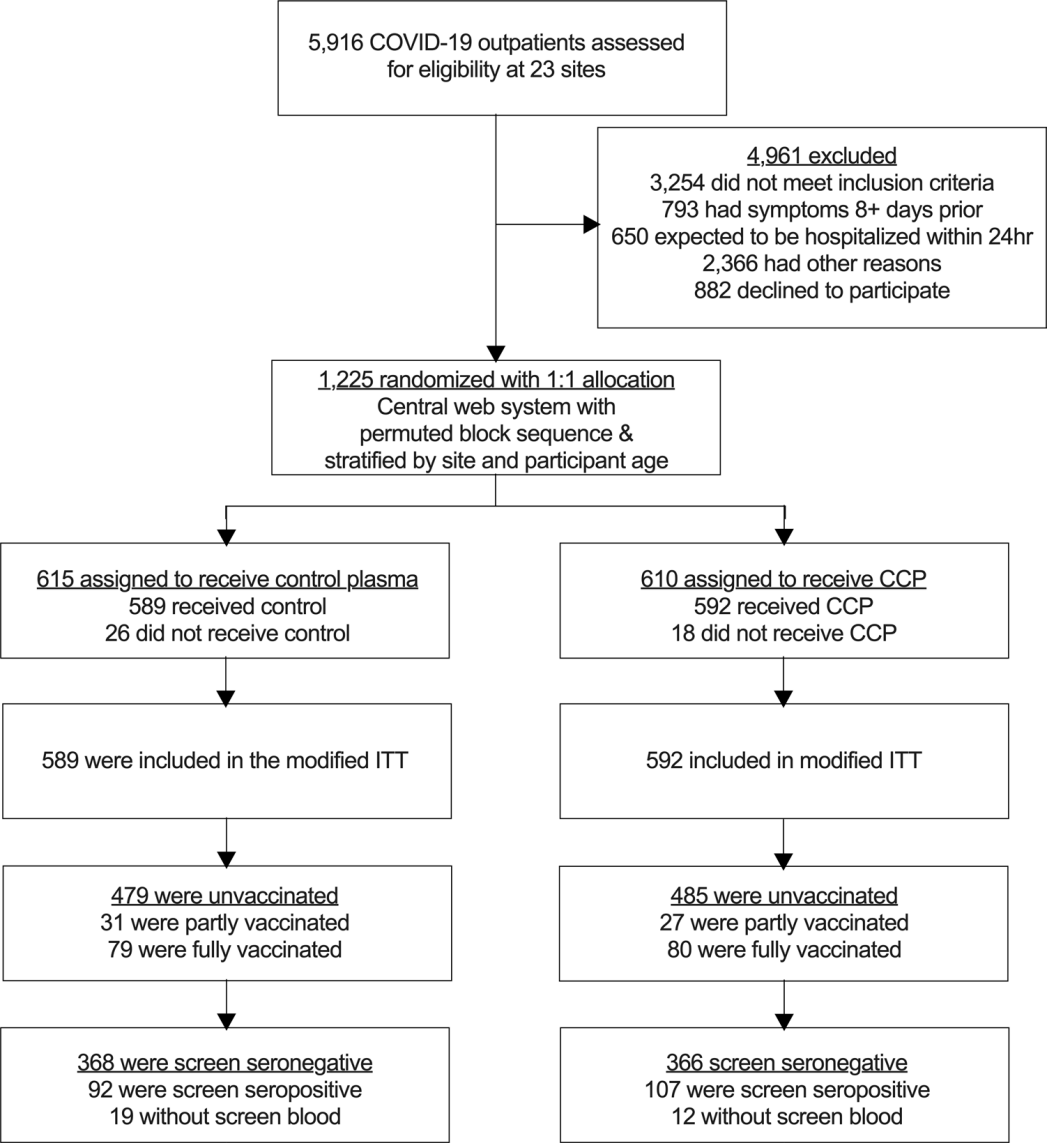
CONSORT diagram depicting enrollment, allocation, and analytical flow of recipients.

This secondary analysis includes the unvaccinated-at-screening subgroup to correlate donor and posttransfusion antibody levels with hospitalization. Transfusions spanned 16 months, from June 3, 2020 to Oct 1, 2021, with the last 3-month follow-up after transfusion in January 2022. The unvaccinated seropositive rate was 21%. Among the seronegative, unvaccinated patients analyzed, 368 received control plasma and 366 received CCP, with an average age of 44 years old. Both control and CCP arms were predominately female, obese (44% with BMI ≥ 30), and had at least 1 preexisting comorbidity (41%). All COVID-19–related hospitalizations in the CCP arm recipients (17 total) were among unvaccinated recipients — 15 seronegative (88%) and 2 seropositive recipients (12%) (Figure 1 and Table 1). Excluded from this analysis were the 159 fully vaccinated with no hospitalizations, 58 partly vaccinated with 1 hospitalization, and 199 unvaccinated seropositive with 7 hospitalizations.

### CCP donor antibody levels.

**Figure 2:**
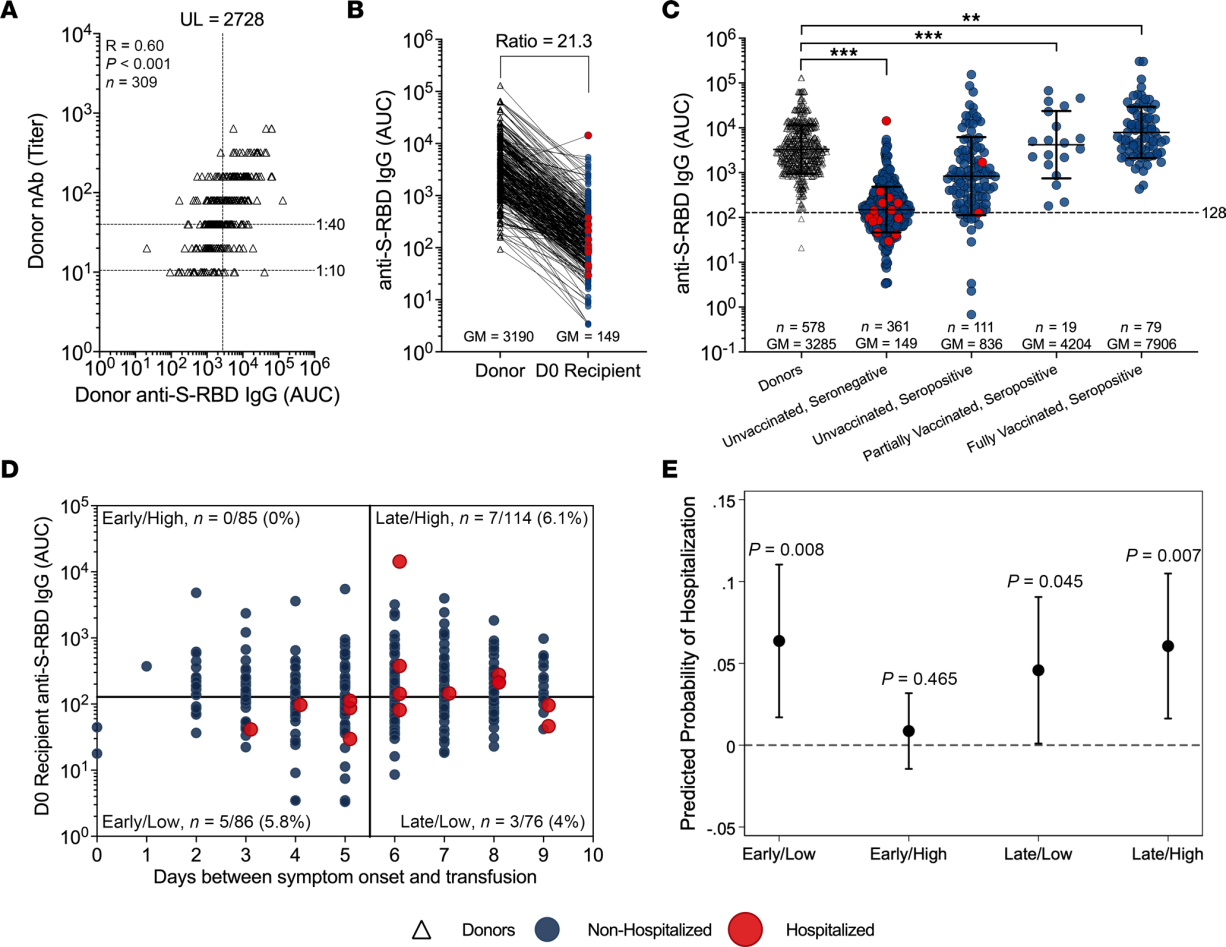
CCP donor neutralizing antibody and anti–S-RBD levels establish a functional cutoff associated with hospitalization protection in screened seronegative recipients. (**A**) Here, we use the 1:40 dilutional titer for the neutralizing antibody (nAb) to identify the upper limit of donor anti–S-RBD IgG 2,728 AUC associated with protection from hospitalization. A dilutional titer of 1:10 is the limit of detection for the nAb. (**B**) The ratio of matched donor anti–S-RBD IgG AUC to that of their respective CCP seronegative recipients that was used to infer the functional cutoff in recipients was determined to be 21.3. Red dots correspond to those hospitalized and black dots are those not hospitalized. (**C**) Anti–S-RBD IgG AUC levels among donors and posttransfusion recipients segregated by screen vaccination status and serostatus compared by Kruskall-Wallis with Dunn’s post hoc correction. **P < 0.002, ***P < 0.001. Unvaccinated subsequently hospitalized (red dots) posttransfusion recipients in screen seronegative (*n* = 15) and screen seropositive (*n* = 2). Black dots are donors and blue dots are nonhospitalized participants. (**D**) Screen seronegative, unvaccinated recipient D0 (posttransfusion) antibody (*n* = 361) segregated by recipient days from symptom onset to transfusion and high (>128 AUC) or low (≤128 AUC) anti–S-RBD IgG levels. Recipient high and low cutoffs were calculated using a 21.3-fold drop from donor anti–S-RBD AUC (upper value of the 95% confidence interval) at a 1:40 nAb titer associated with protection. Subsequently hospitalized (red dots) and nonhospitalized (blue dots) recipients are shown. The *n* values and percentages in each quadrant are the proportion hospitalized among quadrant total. (**E**) Predicted probabilities of hospitalization across early versus late and high (>128 AUC) versus low (≤128 AUC) anti–S-RBD IgG categories of screen seronegative, unvaccinated CCP recipients were compared using Firth’s logistic regression model adjusted for age, sex, BMI, and variant. *P* values that the predicted probability is greater than 0% (horizontal dashed line) are shown, with *P* < 0.05 considered significant.

Approximately 40% of all potential CCP research study donors in the parent study were excluded due to low antibody levels. Unique transfusion units represented the upper 60% of all CCP pre-Delta donors, with a GM anti–Spike receptor-binding domain (anti–S-RBD) IgG titer of 1:6,741 (3,161 AUC). Donor plasma showed strong correlations between anti–S-RBD IgG and virus nAb in dilutional titer and AUC (Figure 2A), as well as donor virus–specific anti–S-RBD IgG antibodies in ng/mL with anti–S-RBD IgG AUC (Supplemental Figure 1; supplemental material available online with this article; https://doi.org/10.1172/jci.insight.178460DS1). We estimate that the total virus-specific anti–S-RBD IgG dose from donor into recipient is 1.2 mg based on a transfusion volume of 200 mL with a donor anti–S-RBD IgG GM of 5.1 μg/mL (200 mL × 5.1 μg/mL = 1,200 μg), indicating recipients have low posttransfusion antibody levels based on current dosing recommendation for CCP (Supplemental Figure 1 and Supplemental Table 1).

### Screen and posttransfusion antibody levels among unvaccinated, seronegative recipients.

The dilution factor associated with the administration of approximately 200 mL CCP was determined by comparing the GM of anti–S-RBD IgG AUC levels of matched donors to that of unvaccinated seronegative recipients. The donor anti–S-RBD IgG AUC GM of 3,190 proportionately decreased by a factor of 21.3 when compared with the anti–S-RBD IgG AUC GM (to 149) for seronegative recipient AUC within 30 minutes of transfusion (Figure 2B). Similarly, 15 seronegative hospitalized CCP recipients had posttransfusion antibody levels 19 times lower than matched donors. The hospitalized and nonhospitalized unvaccinated, screened seropositive CCP participants had a posttransfusion GM anti–S-RBD-IgG AUC of 836, with those partly vaccinated at 4,204 AUC and those fully vaccinated breakthrough infection at 7,908 AUC (Figure 2C). The pretransfusion antibody levels of unvaccinated seropositive participants increased with the days from symptom onset to transfusion (Supplemental Figure 2).

### Posttransfusion recipient antibody benchmarks associated with hospitalization.

Among seronegative control recipients, 8.4% (31/368) were hospitalized, which was higher than the 6.3% hospitalization rate among controls of the parent study that included vaccinated (full and partial) and nonvaccinated, seropositive participants. Hospitalizations in all seronegative CCP recipients were 4.1% (15/366), slightly higher than the full study finding of 2.9%.

**Figure 3:**
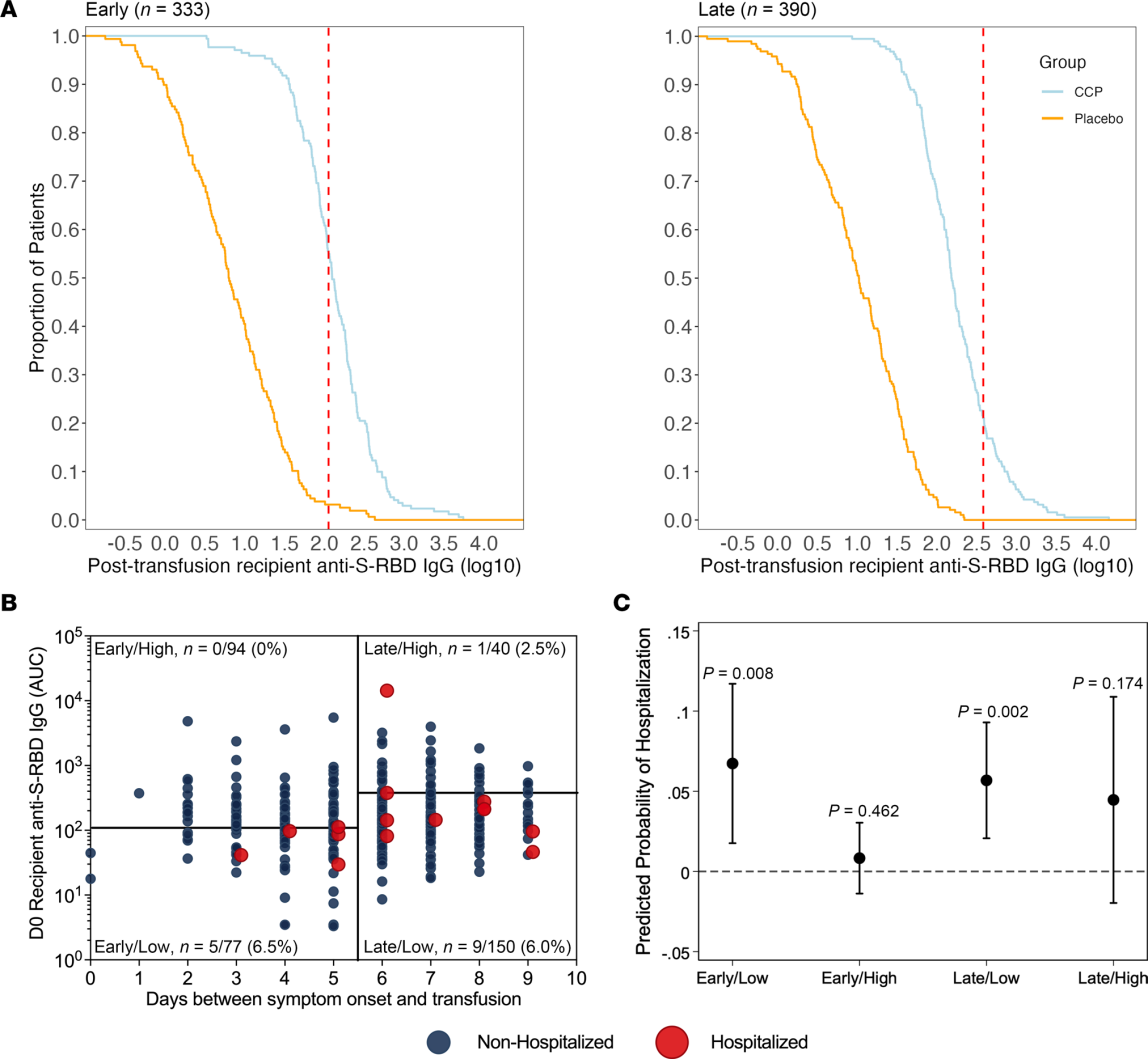
CCP recipient D0 posttransfusion and matched donor antibody levels stratified by duration from symptom onset to transfusion using cutoffs established by the ROC and maximum antibody threshold method. (**A**) RCDCs illustrating antibody distribution of early and late CCP recipients and placebo controls, and thresholds (red dashed lines), established by the maximum antibody that best distinguished hospitalized from nonhospitalized cases. Early recipients are delineated at 2.06 log_10_(anti–S-RBD AUC) (115 AUC), while late recipients are delineated at 2.58 log_10_(anti–S-RBD AUC) (380.2 AUC). Curves exclude 5 early participants and 1 late control participant whose posttransfusion plasma was not available. The *n* values shown are CCP recipients (*n* = 171 early, *n* = 190 late) plus placebo recipients (*n* = 161 early, *n* = 200 late). (**B**) Screen seronegative, unvaccinated recipient D0 posttransfusion antibody (*n* = 361) segregated by early versus late administration assessed as days from symptom onset to transfusion and high versus low antibody using early/late stratum-specific cutoffs established by the maximum antibody that best distinguished hospitalized from nonhospitalized cases. Subsequently hospitalized (red) and nonhospitalized (blue) recipients are shown. The *n* values and percentages in each quadrant are the proportion hospitalized among quadrant total. (**C**) Predicted probabilities of hospitalization across early versus late and high versus low categories among screen seronegative, unvaccinated CCP recipients estimated using Firth’s logistic regression adjusted for age, sex, BMI, and variant. *P* values that predicted the probability is greater than 0% (represented by the horizontal dashed line) are shown for each category, with *P* < 0.05 considered significant.

For this subgroup analysis, we estimated the antibody threshold levels correlated to protection from hospital progression in the CCP group for early and late transfusions — one based on binding antibody levels associated with functional virus neutralization (Figure 2) and another by reverse cumulative distribution curve (RCDC) analysis (Figure 3). For the functional cutoff based on virus nAb, we used a 40-fold dilution of virus nAb, like the correlate of infection protection previously reported for influenza (8). By plotting donor anti–S-RBD-IgG AUC against increasing 2-fold viral dilutions, we identified donor anti–S-RBD IgG 2,728 AUC as the upper limit of the 95% confidence interval of the GM at a 40-fold nAb titer (Figure 2A). After a 21.3-fold dilution, the posttransfusion threshold was calculated to be 128 AUC in recipients. These functional cutoffs delineate high versus low anti–S-RBD IgG levels at 2,728 and 128 AUC for donors and their matched unvaccinated, seronegative recipients, respectively. Recipient posttransfusion antibody levels were plotted by days between symptom onset to transfusion to correlate the functional cutoffs with hospitalization outcome (Figure 2, D and E).

### Virus neutralization–based correlate of protection from hospitalization in recipients.

We observed zero hospitalizations among recipients transfused early (i.e., ≤5 days after symptom onset), with posttransfusion anti–S-RBD IgG levels above 128 AUC as compared with the other 3 CCP quadrants. Although the probability of hospitalization was lowest among recipients receiving early transfusion and high antibody levels above 128 AUC, this group did not reach statistical significance when compared to the other quadrants by Firth’s logistic regression, potentially due to smaller sample sizes (Figure 2E and Supplemental Table 2). Exploratory analysis with Fisher’s exact test revealed a significant association between early/high transfusion, as defined by the nAb-based method, with hospitalization status among other unvaccinated, seronegative CCP recipients (*P* = 0.03), indicating a difference in probability of hospitalization between those with early/high CCP transfusion (0/85, 0%) and those early/low or late CCP (15/276, 5.6%). The early/high CCP compared with all controls (28/368, 7.6%; *P* = 0.004) or early controls (18/167, 11.7%; *P* = 0.0005) was even more significant (Supplemental Table 3).

### ROC-based correlate of protection from hospitalization in recipients.

As an alternative method for identifying antibody thresholds for early recipients, ROC analysis with maximum percentage hospital reduction was used to determine the antibody threshold level for late transfusions. The red dotted line in RCDCs demarcates early-transfusion ROC 115 anti–S-RBD IgG AUC maximum, while the late-transfusion 380 AUC maximized hospitalization difference (Figure 3A). Hospitalization was reduced (0 of 94 hospitalized), with anti–S-RBD IgG 115 AUC (log_10_ of 2.06), while for recipients treated after 5 days from symptom onset, the antibody level for similar treatment efficacy (1 of 40) was anti–S-RBD IgG 380 AUC (log_10_ of 2.58; Figure 3B and Supplemental Table 4). A Firth’s logistic regression comparing CCP quadrants revealed that recipients receiving early transfusion with high posttransfusion antibody levels above anti–S-RBD IgG 115 AUC had the lowest probability of hospitalization, but this difference from other quadrants was not statistically significant (Figure 3C and Supplemental Table 2). Exploratory analysis with Fisher’s exact test revealed a significant association between early/high transfusion, as defined by the RCDC-based method, with hospitalization status among unvaccinated, seronegative CCP recipients (*P* = 0.01), indicating a difference in probability of hospitalization between those with early/high transfusion (0/94, 0%) and those early/low or late CCP (15/267, 5.4%). The ROC early/high CCP compared with all controls (28/368, 7.6%; *P* = 0.002) or early controls (18/167, 11.7%; *P* = 0.0005) had greater significance (Supplemental Table 3).

### Donor antibody–based correlate of protection from hospitalization.

The early/high quadrant for donor plasma units based on the 2,768 AUC (1/88, 1.1%) was also found to be significantly different by Fisher’s exact test from all seronegative controls (31/368, 8.4%) (*P* < 0.002) and early seronegative controls (20/167, 11.9%) (*P* < 0.002; Supplemental Table 3). Donor plasma antibody–based relative risk reduction was 86% and absolute risk reduction was 6.5%. Comparison of donor early/high units to both early/low and late CCP was not significant (Supplemental Table 3).

### Nasal SARS-CoV-2 viral RNA copies at screening.

Nasal viral load might independently determine risk of hospitalization. All unvaccinated individuals subsequently receiving either control plasma or CCP had indistinguishable screen (before plasma transfusion) nasal viral loads, regardless of subsequent hospitalization outcome (Table 1 and Supplemental Figure 3, A and B). Nasal viral loads of those receiving early transfusions were associated with higher viral loads compared with late transfusions, regardless of serostatus at screening or intervention (Supplemental Figure 3C). Pre-Delta viral loads segregated by seronegative or seropositive and days from symptom onset to screen showed a decrease in viral load by day, with a sharper drop after day 5 from symptom onset (Supplemental Figure 3D). Delta-period viral loads in unvaccinated and vaccinated individuals showed a similar drop in viral load with later transfusions (Supplemental Figure 4). While our inclusion criteria required a documented positive molecular SARS-CoV-2 test (87% by RNA detection and 13% by antigen detection), the interval between subjects’ initial pre-enrollment SARS-CoV-2 test and our pretransfusion nasal swab may have been up to 7 days.

### Longitudinal antibody kinetics following transfusion.

**Figure 4:**
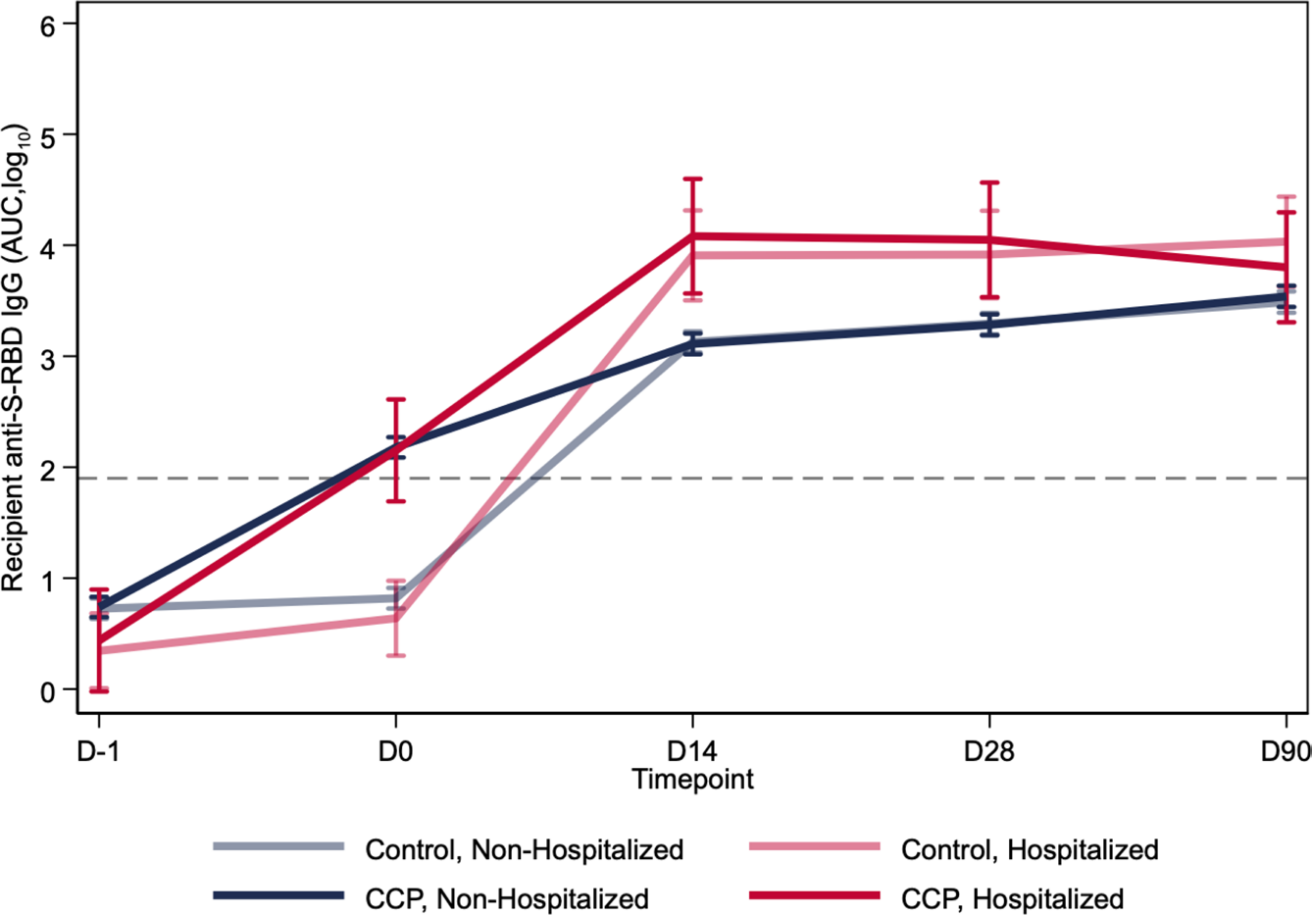
Antibody levels over 3 months after transfusion by hospitalization status and treatment group for screen seronegative, unvaccinated recipients. Log_10_-transformed antibody levels up to 90 days after transfusion were segregated by treatment and hospitalization status of recipients using a linear mixed-effects regression model, adjusted for variant, age, sex, and BMI. CCP recipients have greater AUC levels on D0, but by D14, the hospitalized recipients have greater AUC levels than nonhospitalized. The average time from transfusion to hospitalization was 3.05 days, with all posttransfusion hospitalizations occurring between D0 and D14. The dashed line represents the log-transformed cutoff (1.924 AUC) for seropositivity. This diagnostic threshold is equivalent to the anti–S-RBD IgG log_10_-transformed 180 titer.

Antibody levels at or beyond 14 days after transfusion did not differ between CCP and control plasma recipients (Figure 4). Hospitalization status, but not treatment, affected antibody levels over time. The multivariable linear mixed-effects regression, adjusted for variant, age, sex, and BMI, showed no differences in antibody levels between CCP and control plasma recipients beyond 14 days after transfusion (Figure 4 and Table 2). There were no sex, age, BMI, or comorbidity differences in antibody levels between CCP and control groups. At the day 90 follow-up visit, anti–S-RBD IgG AUC levels were similar for control and CCP and increased during the pre-Alpha, Alpha, and Delta variant periods, as well as among fully vaccinated recipients (Supplemental Figure 5).

## Discussion

In this secondary analysis of our outpatient, double-blind, placebo-controlled trial of CCP to prevent hospitalizations, we documented that donor CCP in the top 30% by anti-S antibody levels increased seronegative recipient antibody thresholds to sufficiently high cutoffs that when administered early within 5 days of symptom onset were effective in hospital reduction. Initial screen nasal viral loads did not impact hospital outcome.

At the start of the pandemic, there were no evidence-based standards for CCP donor antibody levels. Most of the donor emergency use authorization (EUA) qualification of high titer after February 2021 was based on anti-S antibody levels rather than neutralizations. Diversity in methods used antibody quantification and the need for harmonization of assays across institutions became apparent (16, 17). Within our study, the donor binding anti–S-RBD IgG of 2,728 AUC corresponded to live virus neutralization of greater than 1:40 in donors, and if transfused within 5 days of COVID-19 symptom onset, reduced hospitalization. Initially, the FDA recommended donor plasma qualification for the outpatient CCP study under IND 19725 as seropositive after a 1:320 dilution (5). CCP donors for the hospitalized Expanded Access Program from March to August 2020 in the United States reported more than 10-fold higher median virus neutralization, using the Broad Institute plaque reduction neutralization test (D614G) of 1:525 (2). The outpatient C3PO study used a microneutralization assay with a median titer of 1:578 (18). The Argentina outpatient study used a CCP cutoff of 1:84 based on a surrogate virus neutralization test and segregating to the upper half of donors improved outcome (19). The effective CONFIDENT trial used CCP with virus neutralization of greater than 1:160, representing the top 15% of Belgium donors in the pre-Delta time periods in those hospitalized and newly mechanically ventilated (14). While the lack of standardization impedes comparative virus neutralization analysis, all studies highlight that utilizing donors with high-titer virus nAbs is critical for CCP effectiveness.

When CCP was first deployed in 2020, there were concerns that specific antibody administration to individuals in the early stages of COVID-19 could interfere with the development of endogenous immune responses (20). However, our findings show that transfusion of CCP, as compared with control plasma, was not associated with differences in the total antibody-level immune response in recipients with convergence by day 14, which is reassuring for the immunological safety of CCP in humans. The C3PO convalescent plasma study also demonstrated no antibody level difference between CCP and saline infusions (18, 21).

Strengths of this study include the large population of 1,181 participants, well-characterized donor and recipient antibody levels measured by diverse metrics, and overall trial effectiveness in hospital reduction that extended to subpopulations at risk of severe disease progression like diabetes, hypertension, obesity, and increasing age. Limitations of the study include predominately SARS-CoV-2–naive recipients enrolled prior to the Omicron variant who were largely unvaccinated such that the findings are only approximately applicable to immunocompromised patients or others who lack SARS-CoV-2 antibodies. Another limitation is the low number of seronegative participants transfused within 5 days of symptom onset, with posttransfusion donor antibody levels above the GM in our study population (approximately 100 participants). The study randomized participants to CCP and control plasma, not early or late transfusions stratified by antibody level. The parent study was not powered to look at these stratified quadrants. While the influenza titer was set by the WHO at 1:40, this correlate of hospital protection still needs to be established for SARS-CoV-2 for different phases of COVID-19.

Our results provide evidence for the best use of CCP. In summary, our results support and confirm that for antibody therapy to be effective, sufficient levels of pathogen-specific antibodies need early administration (15). The retrospective virus-specific antibody levels measured in 19,000 donors used in the Convalescent Plasma Expanded Access Program measured anti-RBD antibodies at 54 μg/mL, translating to 10 mg/200 mL for the BARDA study (2). The mass amount of virus-specific antibody needed for outpatient CCP efficacy (1.2 mg) in this study was 10-fold lower than that in the EAP study and, importantly, lower (100- to 1000-fold) than when mAbs were used at 150 mg to 2,100 mg total IgG dose, which may reflect convalescent plasma synergy between the many antibody specificities and isotypes in the polyclonal response, which bind multiple epitopes, cooperate in neutralization, and utilize diverse constant region functionality. One milliliter of plasma has 11 mg/mL total IgG antibody, translating to 200 mL with 2,200 mg, or 2.2 g, of IgG. The average donor virus–specific anti–S-RBD IgG of 1.2 mg is less than 0.1% of 2.2 g. No hospitalizations were observed in those recipients treated within 5 days of symptom onset with these high antibody levels, indicating that this is the optimal dose and timing combination for effective CCP use. Early treatment alone is insufficient, as hospitalizations were still observed in the group treated within 5 days with lower titer units, necessitating both early treatment and adequate antibody dosing for optimal efficacy.

Although our results are less relevant to COVID-19 in the fourth year of the pandemic when the majority of immunocompetent individuals have endogenous antibody from vaccination and/or infection, they are highly relevant to both the currently immunocompromised COVID-19 patients without functional SARS-CoV-2–specific antibodies or to future deployments of convalescent plasma for infectious disease emergencies. We advocate that CCP units reserved for therapy comprise greater antibody levels restricted to the upper 20%–30% of all donors to protect against future variants (22–24) or a novel microbe. Doubling the volume to nearly 500 mL with 2 units of approximately 210 mL also increases antibody levels along with increasing titer.

When humanity faces its next pandemic, there is a high likelihood that convalescent plasma will be used again until better specific therapies become available. Our data provide a roadmap for optimal early, high-dose (upper deciles) convalescent plasma deployment in future emergencies.

## Methods

### Sex as a biological variable.

Our study examined male and female participants, with statistical comparisons between the sexes. Similar findings are reported for both sexes.

### Study design.

This study is a follow-up secondary analysis to correlate donor and recipient antibody levels to hospital outcome within a large outpatient, double-blind, randomized clinical trial comparing CCP to control plasma at 23 centers throughout the United States from June 2020 through September 2021 (5). Symptomatic adults (≥18 years old) with a confirmed SARS-CoV-2–positive test, regardless of vaccination status or risk factors for severe COVID-19, were enrolled within 8 days of symptom onset. Over 5,000 recipient plasma samples were collected at pretransfusion screening (D-1), 30 minutes after transfusion (D0), and follow-up visits (D14, D28, D90) (25). This subgroup analysis was restricted to seronegative, unvaccinated CCP recipients. CONSORT reporting guidelines were utilized (26). Detailed procedures are in the Supplemental Methods.

### Study donor plasma.

The study qualified donor plasma with SARS-CoV-2–positive antibodies after a 1:320 dilution under the FDA IND 19725 protocol. After July 2021, the transfused plasma donor units met the existing FDA EUA criteria for high titer at a EUROIMMUN arbitrary unit (AU) over 3.5. These donor units were previously characterized for full-length anti-S IgG GM titers of 13,053, which corresponded to a more precise AUC GM of 7,938, equivalent to 243 BAU/mL using the international standards (5). The median nAb titer was 80, with a GM titer of 58, and nAb AUC of 51, equivalent to GM 27 IU/mL (5). The commercial EUROIMMUN AU mean was 6 for the unique donor units (5).

### Indirect anti–S-RBD ELISA.

The anti–S-RBD ELISA was adapted from a published protocol (27). The anti–S-RBD IgG threshold for seronegativity was 180 titer or lower. Serostatus was determined based on screening antibody levels. The seropositive anti–S-RBD IgG ELISA titers represent 3-fold dilutions from 540 to 393,660. Anti–S-RBD IgG dilutional titer and AUC were quantified. The limit of detection (LOD) was calculated to be half of the lowest AUC for samples with detectable titer (≥1:20) and samples with undetectable titers (1:10) were set be half the LOD. The 96-well plates (Immulon 4HBX, Thermo Fisher Scientific, 3855) were coated with anti–S-RBD of the parent strain at a volume of 50 μL of 2 μg/mL diluted antigen in filtered, sterile 1× PBS (Thermo Fisher Scientific) at 4°C overnight. The coating buffer was removed, and the plates were washed 3 times with 300 μL of 1× PBS plus 0.1% Tween 20 (PBST) wash buffer (Thermo Fisher Scientific) and then blocked with 200 μL PBST with 3% nonfat milk (milk powder, American Bio) by volume for 1 hour at room temperature. All plasma samples were heat inactivated at 56°C on a heating block for 1 hour before use and diluted 1:2 in PBS. Negative control samples were prepared at 1:10 dilutions in PBST with 1% nonfat milk and plated at a final dilution of 1:100. A mAb against the SARS-CoV-2 S protein was used as a positive control (1:5,000 dilution; Sino Biological, 40150- D001). Plasma samples were prepared in 3-fold serial dilutions starting at 1:20 in PBST with 1% nonfat milk. Blocking solution was removed, and 100 μL diluted plasma was added in duplicate to the plates and incubated at room temperature for 2 hours. Plates were washed 3 times with PBST, and 50 μL of secondary antibody was added to the plates and incubated at room temperature for 1 hour. Antihuman secondary antibody, Fc-specific total IgG HRP (1:5,000 dilution; Thermo Fisher Scientific, Invitrogen, A18823), was prepared in PBST plus 1% nonfat milk. Plates were washed, and all residual liquid was removed before the addition of 100 μL SIGMAFAST OPD (*o*-phenylenediamine dihydrochloride) solution (MilliporeSigma) to each well, followed by incubation in darkness at room temperature for 10 minutes. To stop the reaction, 50 μL of 3 M HCl (Thermo Fisher Scientific) was added to each well. The OD of each plate was read at 490 nm on a SpectraMax i3 ELISA Plate Reader (BioTek Instruments). The positive cutoff value for each plate was calculated by summing the average of the negative values and 3 times the SD of the negatives. LODs were set to half the lowest AUC value at or below 20 titer. The anti–S-RBD IgG titer threshold for seronegative was 180 titer or below. The seropositive anti–S-RBD IgG ELISA titers represent 3-fold dilutions from 540 to 393,660.

### Quantification of virus-specific anti–S-RBD and anti–full-length S in ng/mL.

Quantitative antibody measurements were based on an electrochemical immunoassay protocol as previously published (28). A fusion protein of anti–human IgG coupled with 2 invertases was used as the electrochemical reporter. Antibody concentrations in ng/mL were obtained by measuring the amount of glucose generated by the protein fusion during immunoassays, based on quantitative dose-response curves built using commercial anti-RBD, anti–N-terminal domain (anti-NTD), and anti-S2 antibodies. The protocol was adapted to run on a 96-well plate (Nunc, Thermo Fisher Scientific, 262162). Each well was coated using 50 μL of either S-RBD or full S protein in PBS, at concentrations of 2.5 and 5.0 ng/mL, respectively. The coating was conducted overnight at 4°C. Wash buffer (WB) was prepared with 1× PBS, pH 7.4 (Fisher Chemical) plus 0.05% Tween (Fisher Bioreagents). Blocking buffer (BB) was prepared by dissolving casein (Fisher Chemical) at 5% w/v in WB. The incubation temperature for each step after coating was 25°C. After coating, the plates were washed 3 times with WB and then blocked with 200 μL BB for 1 hour. Then, the plates were washed 3 times with WB. This procedure was followed by a 30-minute incubation with 50 μL of patient plasma specimens diluted to 1% or 20% with BB, depending on titer levels. Each specimen was interrogated in triplicate. Positive controls (125 and 1,000 ng/mL) and calibration curves (0 to 5,000 ng/mL) for the S-RBD assay employed a commercial mAb against SARS-CoV-2 S glycoprotein S1 (Abcam, ab273073) prepared in 1% or 20% control plasma (to account for both dilutions) diluted in BB. For the full-length S protein assay, a 1:1:1 mAb mix against SARS-CoV-2 S glycoprotein S1 (Abcam, ab273073), SARS-CoV-2 S2 (Novus Biologicals, NBP3-07956), and SARS-CoV-2 S NTD (ACROBiosystems, SPD-S164) was diluted similarly as for the S-RBD alone. After specimen incubations and washing 3 times with WB, 50 μL of 0.02 μM LC15 antibody-invertase fusion protein in BB was added and incubated 1 hour. The plates were washed 3 times with WB and once with 1× PBS, pH 7.4. This was followed by a 1-hour incubation with 50 μL of 100 mM sucrose (Fisher Chemical) in 1× PBS, pH 5, with glucose concentration measured immediately after using a medical-grade glucometer (Nova Biomedical). Calibration curves were analyzed via nonlinear regression of the Hill isotherm (Igor Pro 8 software) and used to calculate the antibody concentration from the average glucose concentration of each plasma sample.

### SARS-CoV-2 viral copy quantification.

Nasopharyngeal specimens obtained at screening were stored in 5 mL of virus transport media at –70°C on site, and then shipped to the central storage facility at Johns Hopkins University. RNA was extracted from 200 μL transport media with either the Qiagen viral RNA extraction kit or the Chemagic Viral DNA/RNA 300 kit 96 (Perkin Elmer) followed by real-time reverse transcriptase quantitative PCR (RT-qPCR) assays targeting the SARS-CoV2 nucleocapsid (N) gene and the human RNaseP gene using methods described by the US CDC (29).

### SARS-CoV-2 virus neutralization assay.

Plasma nAbs were determined against WA-1 (SARS-CoV-2/USA-WA1/2020 EPI_ISL_404895), obtained from BEI Resources, as described previously (30, 31). The limit of viral neutralization detection was at 1:10 titer.

### Statistics.

The comparative analysis of anti–S-RBD IgG antibody levels involved calculating the ratio between unique CCP donors and posttransfusion seronegative, unvaccinated recipients. This calculation was performed by dividing the GM of the AUC values for donor samples by the corresponding AUC values for the CCP recipients.

We determined correlates of protection based on donor anti–S-RBD IgG levels using 2 methods: one relying on virus neutralization and the other employing ROC curve analysis. In the first approach, we established a functional cutoff value for binding antibody levels through virus neutralization to distinguish between high and low donor anti–S-RBD IgG AUC levels. It is noteworthy that a virus nAb at a 1:40 dilutional titer has been previously identified as a correlate of protection in influenza studies (6–8). Initially, we computed the upper limit of the 95% confidence interval for the donor anti–S-RBD IgG AUC GM, corresponding to a donor nAb at a 1:40 dilutional titer. The GM was found to be 2,291 AUC, with a lower limit of 1,924 and an upper limit of 2,728 AUC. Considering that the antibody levels of seronegative CCP recipients were approximately 21.3 times lower than those of their respective donors, we extrapolated the functional cutoff point for CCP recipients to be 21.3 times lower than that of donors, resulting in a value of 128 AUC.

RCDCs were plotted (32) for control and CCP recipient anti–S-RBD posttransfusion ROC analysis. An estimated optimal threshold value from the ROC curve maximizing sensitivity and specificity determined the antibody threshold level for early transfusion. For late transfusions, the maximal percentage hospital reduction defined the antibody threshold level.

Spearman’s correlations were used to evaluate strength of association between titer and AUC units for antibody measurements. The predicted probabilities of hospitalization based on early versus late and high versus low categories were assessed using Firth’s logistic regression model, chosen due to complete separation in the data set. Statistical association between hospitalization status and early/high transfusion was assessed by Fisher’s exact test. Comparisons across groups were performed using the Kruskall-Wallis multiple-comparison test with Dunn’s post hoc corrections. We analyzed the antibody kinetics over time among unvaccinated, seronegative recipients using a linear mixed-effects regression model, adjusted for variant, age, sex, and BMI, with anti–S-RBD IgG log_10_(AUC) data. An interaction term was included to examine how antibody levels changed over time by treatment (control or CCP) and hospitalization status. Predicted effects were graphed with 95% confidence intervals. *P* values less than 0.05 were considered statistically significant. Analyses were performed using Prism 8 (GraphPad Software) or Stata 17 (StataCorp).

### Study approval.

Johns Hopkins University served as the single IRB. For the Center for American Indian Health sites, the protocol was also independently reviewed and approved by the Navajo Nation Health Human Research Review Board and the Indian Health Service IRB. The protocol was also approved by the Department of Defense Human Research Protection Office. The trial was conducted in accordance with the principles of the Declaration of Helsinki, the Good Clinical Practice guidelines of the International Council for Harmonization, and all applicable regulatory requirements. Written and signed informed consent was obtained from all participants. The trial was registered in ClinicalTrials.gov (NCT04373460).

### Data availability.

Data are available from authors upon request, with reply expected in 14 days. All data within graphs are contained within the Supporting Data Values file. Deidentified data from clinical trials has been deposited in the Vivli server (compliant with General Data Protection Regulations) for public access. Users either can access the Vivli data by downloading it or have access to a remote desktop workspace in a secure virtual research environment (https://vivli.org/resources/requestdata/). 

### Code availability.

Unique software or computational code was not created for this study.

## Author contributions

HSP, CB, A Yin, JSL, CAC, YE, REF, ORB, JS, TJG, PC, JW, NN, MJB, AART, OL, SLK, and DJS contributed to the experimental design and procedures for the anti–S-RBD antibody level measurements. KL, NAM, DEF, LJA, BL, DFH, AC, SS, EMB, KAG, AART, OL, AP, SLK, and DJS conceived and/or designed the clinical work. ML, SY, IS, AJ, and AP contributed to the experimental design and procedure for the viral load and neutralizing antibody measurements. EOG and NAC performed and supervised, respectively, glucometer-based antibody quantification measurements. AGS, GSM, YF, BP, SLH, ACL, BRM, ESS, SA, MAH, JEB, JSC, JHP, JMG, JRP, PBB, WR, MEC, JH, BG, VCC, DC, KO, MA, LLH, CGS, DNF, MSZ, ERC, JSR, SGK, CEM, MR, A Yarava, KL, NAM, ALG, NK, AS, DEF, DAJ, LJA, DMS, BL, SE, SNB, TJG, AZ, DFH, AC, SS, EMB, KAG, AART, OL, AP, SLK, and DJS conducted/contributed to the clinical study and/or collected clinical data. HSP, CB, A Yin, JSL, CAC, ML, SY, IS, AJ, MR, REF, ORB, JS, JO, YBN, MDK, TJG, PC, DFH, AC, KAG, AART, OL, AP, SLK, and DJS contributed to data processing and analyses specific to this work. HSP, CB, A Yin, AC, AP, SLK, and DJS drafted the manuscript. All authors provided final approval of the version to be published. The order of the co–first authors was determined based on role in executing experiments, analyses, and manuscript writing.

## Supplementary Material

Supplemental data

ICMJE disclosure forms

Supporting data values

## Figures and Tables

**Table 1:**
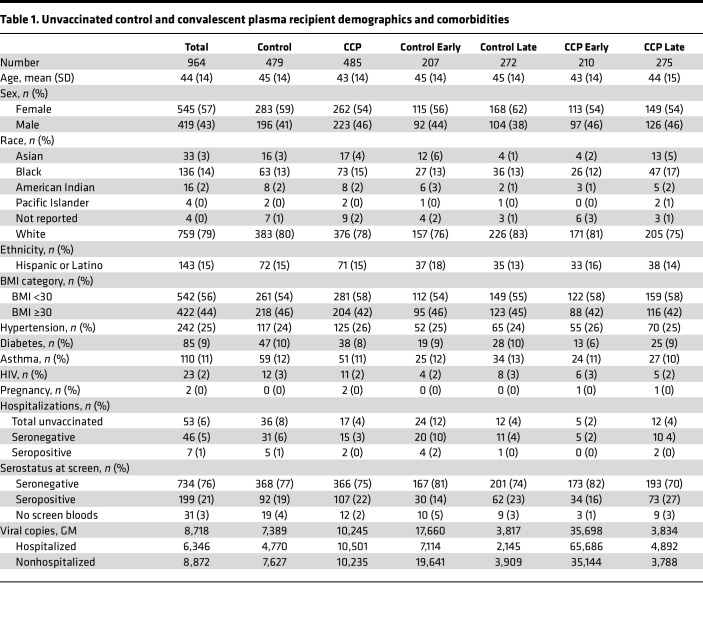
Unvaccinated control and convalescent plasma recipient demographics and comorbidities

**Table 2:**
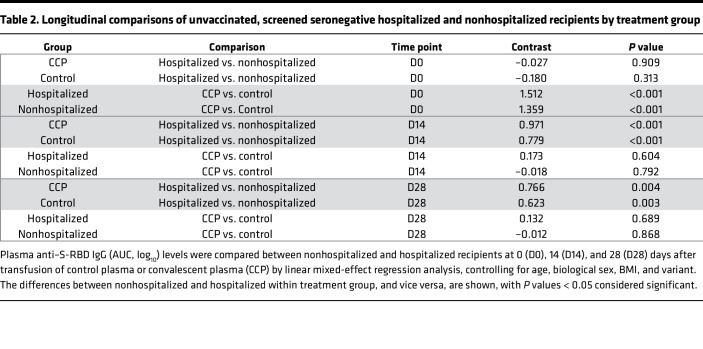
Longitudinal comparisons of unvaccinated, screened seronegative hospitalized and nonhospitalized recipients by treatment group

## Version 1 03/14/2024

In-Press Preview

## Version 2 04/22/2024

Electronic publication
