# Supplementary material for: Outpatient COVID-19 convalescent plasma recipient antibody thresholds correlated to reduced hospitalizations within a randomized trial
Source: JCI Insight. 2024 Mar 14;9(8):e178460. doi: 10.1172/jci.insight.178460 (PMC11141865; doi:10.1172/jci.insight.178460)
Supplement: Supplemental data [file jciinsight-9-178460-s065.pdf]

Outpatient COVID-19 Convalescent Plasma Antibody Levels Associated with Reduced Hospitalizations: Secondary Analysis of a Randomized Clinical Trial
Supplement Methods figures and tables

Table of contents

Full Methods	2
Study Design, Ethics, Population and Centers	2
Study donor Plasma, Visits and Indirect ELISA	3
Quantification of Viral Specific anti-S-RBD	4
Viral load and Neutralization	5
Statistical Analysis	5
Supplemental Figure 1 Conversion of anti-S-RBD AUC to ng/mL	7
Supplemental Figure 2 Screen seropositive participants antibody levels	8
Supplemental Figure 3 Screen pre-transfusion nasal swab viral load	9
Supplemental Figure 4 Screen viral loads during the Delta period.	10
Supplemental Figure 5 Antibody levels three months post-transfusion	11
Supplemental Table 1 Data for ng/mL glucose based determination	12
Supplemental Table 2 Firths logistic regression probabilities and statistics	13
Supplemental Table 3 Fishers Exact table results-unvaccinated, seronegative	14
Supplemental Table 4 ROC analysis	15
References	16

Full methods

Study design

Our study is a follow-up secondary analysis of a large outpatient, double-blind, randomized clinical trial comparing CCP to control plasma which sought to correlate donor and recipient antibody levels to hospital outcome at 23 centers throughout the United States from June 2020 through September 2021[1]. The trial was halted at 92% (1181/1280) transfusions because of diminishing hospitalizations with increasing vaccinations in fall of 2021. Symptomatic, SARS-CoV-2 test positive, ages 18 or older, regardless of vaccination status or risk factors for severe COVID-19 participants were enrolled within 8 days of symptom onset. The anti-S-RBD IgG dilutional titer and the more precise AUC was quantified in over 5,000 recipient samples at pre-transfusion screening (D-1), 30 minutes post-transfusion (D0), and follow-up visits (D14, D28, D90)[2]. This subgroup analysis was restricted to seronegative, unvaccinated participants. Full study protocol and statistical plan with protocol changes are available with previous publication[1].

Study sample size was calculated to be 1280 at start of study based on recruitment of an 50% older population with estimate of 30% hospitalization and 15% in age less than 65 years. We assume a one-sided Type I error rate (alpha) of 0.05 as we are interested in superiority and Type II error rate (beta) of 0.2. Therefore, with a sample size of 1344 (1280*1.05 to allow for potential losses) with a target of a minimum ratio of 50:50 for <65:≥65 years of age, we expected to detect at least a 25% reduction in the rate of hospitalization under 80% power and a 30% reduction in rate of hospitalization with 90% power.

Study Ethics

Johns Hopkins served as the single-IRB (sIRB). For the Center for American Indian Health sites, the protocol was also independently reviewed and approved by the Navajo Nation Health Human Research Review Board and the National Indian Health Service IRB. The protocol was also approved by the Department of Defense (DoD) Human Research Protection Office (HRPO). An independent medical monitor who was unaware of the trial group assignments reviewed all serious adverse events, and an independent panel of three physicians who were unaware of the trial-group assignments adjudicated Covid 19 related hospitalizations and severity. An independent data and safety monitoring board provided interim safety and efficacy reviews. The trial was conducted in accordance with the principles of the Declaration of Helsinki, the Good Clinical Practice guidelines of the International Council for Harmonisation, and all applicable regulatory requirements. Written and signed informed consent was obtained from all participants.

Study Population

In this multicenter, double-blind, randomized, controlled trial, we evaluated the efficacy and safety of COVID-19 convalescent plasma, as compared with control plasma, in symptomatic adults (≥18 years of age) who had tested positive for severe acute respiratory syndrome coronavirus 2, regardless of their risk factors for disease progression or vaccination status. Participants were enrolled within 8 days after symptom onset and received a transfusion within 1 day after randomization. The primary study outcome (reported previously) was COVID-19–

related hospitalization within 28 days after transfusion. There were no obvious imbalances between the trial groups in the parent trial with respect to baseline characteristics, including coexisting conditions, COVID-19 vaccination status, vital signs, and clinical laboratory results.

Study Center(s)

Anne Arundel Medical Center; Ascada Research; Baylor College of Medicine; Johns Hopkins Center for American Indian Health; Johns Hopkins Bloomberg School of Public Health; Johns Hopkins University; Lifespan/Brown University Rhode Island Hospital; Mayo Clinic, Phoenix; MedStar Washington Hospital Center; NorthShore University Health System; The Bliss Group; The Next Practice Group; University of California, Los Angeles Health; University of Alabama at Birmingham; University of California, Irvine Health; University of California, San Diego; University of Cincinnati Medical Center; University of Massachusetts Worcester; University of Miami; University of New Mexico; University of Rochester; University of Texas Health Science Center at Houston; University of Utah Health; Vassar Brothers Medical Center; Wayne State University; Western Connecticut Health Network, Danbury Hospital; Western Connecticut Health Network, Norwalk Hospital.

Study Donor Plasma

The study qualified donor plasma with SARS-CoV-2 positive antibodies after a 1:320 dilution under FDA IND 19725 protocol. After July 2021, the transfused plasma donor units met the existing FDA Emergency Use Authorization (EUA) criteria for high titer at EUROIMMUN arbitrary unit (AU) over 3.5. Many identical apheresis donor plasma units were transfused into 2, 3, or 4 separate recipients. Plasma from 333 unique CCP donations was transfused into the 592 CCP participants. Seventy-five percent of the donor collections were before September 2020 with more than 90% by January 2021 and the last 25 collections by March 2021. These donor units were previously characterized for full-length anti-Spike IgG geometric mean (GM) titers of 13,053, which corresponded with a more precise area under the curve (AUC) geometric mean of 7938, equaling 243 BAU/mL using the international standards[3]. The median neutralizing antibody (nAb) titer was 80, with a geometric mean titer of 58, and nAb AUC of 51, equaling GM 27 IU/mL[3]. The commercial EUROIMMUN arbitrary units (AU) mean was 6 for the unique donor units[3].

Study blinding and allocation

Blinding- Both investigational products—COVID-19 convalescent plasma and control plasma—were matched for ABO compatibility, and the existing labels were covered with labels that read “Thawed plasma (volume), store at 1–6°C; new drug limited by federal (or U.S.) law to investigational use” in order to preserve verification codes.

Allocation-After screening, participants from all 23 sites were randomly assigned in a 1:1 ratio with the use of a central Web-based system and a permuted-block sequence to receive either CCP or control plasma (each administered in a single dose at a volume averaging 214 mL). Randomization was stratified according to trial site and participant age (<65 years or ≥65 years). The procedures related to randomization of participants at the clinical sites was as follows: Clinical sites collected randomization eligibility and baseline data on the appropriate data collection instruments and entered these data into the database.

The data system confirmed randomization eligibility, issued the next assignment, and relayed treatment assignments to the Data Cordinating Center (DCC) (masked) and blood bank (unmasked).
The data system automatically stored the date and time of assignment, the identity of the clinical site personnel making the assignment, the participant's ID, and the treatment group assignment.

Study visits and time periods
In these studies, antibody levels were measured at screen before transfusion, within 30 minutes of transfusion, and various timepoints up to 90 days post-transfusion. Participants were transfused during pre-Alpha (June 3, 2020 to January 31, 2021), Alpha (February 1, 2021 to July 14, 2021), and Delta (July 15 to October 1, 2021) variant periods. There were just three participants transfused from July 2 to July 9, 2021 which decreased the number of false designations. The first Alpha (B1.1.7) confirmed by sequencing was from a participant transfused February 18, 2021.

SARS-CoV-2 Viral Load
Nasopharyngeal specimens obtained at screen were stored in 5 mL of virus transport media at -70˚C on site, then shipped to the central storage facility at Johns Hopkins University. RNA was extracted from 200 mL transport media with either the Qiagen viral RNA extraction kit (Qiagen, Hilden, Germany), or the chemagic Viral RNA/DNA 300 H96 kit with chemagic 360 nucleic acid extraction system (Perkin Elmer), according to manufacturer recommended protocols. Real-time reverse transcriptase quantitative PCR (RT-qPCR) assays targeting the SARS-CoV2 nucleocapsid (N) gene and the human RNaseP gene were performed based on the methods described by the US CDC[4]. Primer and FAM-labelled probe sets for CDC nCoV_N1 and RNaseP assays were purchased from IDT (Integrated DNA Technologies) as part of the SARS-CoV2 Research Use Only RUO qPCR primer and probe kit (part number 10006713, 2019_nCoV RUO kit). Single-plex assays with equivalent volumes of RNA (or Positive Control, Plasmid-RNA Standards or Nuclease Free H2O for No Template Controls (NTCs)) were performed using the TaqPath 1-Step RT-qPCR MasterMix (Applied Biosystems, ThermoFisher Scientific) in a QuantStudio 5 Real-Time PCR system (ThermoFisher Scientific). The SARS-CoV-2 nCoV-N control plasmid comprised the complete nucleocapsid gene of SARS-CoV-2 isolate Wuhan-Hu-1, complete genome (GenBank: NC_045512.2), and the HsRPP30 Positive control contained a portion of the RNAseP (RPP30) gene. Both plasmid controls were purchased from IDT. Standards for quantitative analysis were prepared from serial dilutions of the nCoV-N and HsRPP30 plasmid controls for which target copy number was known. The range covered was 200,000 copies to 320 copies. Standard curve analysis of nCoV_N1 Ct values was performed by the QuantStudio Design and Analysis software to determine RNA copies of viral genome. Only samples with quantities within the standard curve range were given a COVID-19 call/score "positive". A Ct value for the RNaseP gene was used to verify that human RNA was present in each specimen. For samples that did not amplify viral genome or any host cell RNA, a repeat RT-qPCR was performed and subsequently assigned as "undetermined".

SARS-CoV-2 Virus Neutralization Assay

Plasma neutralizing antibodies were determined against WA-1 (SARS-CoV-2/USA-WA1/2020 EPI_ISL_404895), which was obtained from BEI Resources, as described previously[5,6]. Two-fold dilutions of plasma (starting at a 1:20 dilution) were made and infectious virus was added to the plasma dilutions at a final concentration of 1×10^5 $TCID_{50}$/mL (100 $TCID_{50}$ per 100 µL). The samples were incubated with the virus for 1 hour at room temperature, and then 100 µL of each dilution was added to 1 well of a 96-well plate of VeroE6-TMPRSS2 cells in hexaplicate. The cells were incubated for 6 hours at 37°C, 5% CO_2. The inocula were removed, fresh infection media (IM) was added, and the cells were incubated at 37°C, 5% CO_2 for 2 days. The cells were fixed by the addition of 100 µL of 4% formaldehyde per well, incubated for at least 4 hours at room temperature, and then stained with Napthol Blue Black (MilliporeSigma). The neutralizing antibodies titer was calculated as the highest serum dilution that eliminated the cytopathic effect in 50% of the wells (NT50), and the AUC was calculated using Graphpad Prism.

Primary Study Statistical Analysis

The statistical analysis plan, included with the trial protocol at NEJM.org, was finalized before database lock and unblinding. We calculated the risk difference and the restricted mean survival time (the expected mean time to hospitalization or death by 28 days) in a modified intention-to-treat analysis that excluded participants who did not receive transfusion of convalescent plasma or control plasma. We estimated the cumulative incidence using the doubly robust estimator based on a targeted minimum loss–based estimator. In order to increase the precision of estimates and to account for potential dependent censoring, the analyses were adjusted for baseline variables that were potentially related to the primary outcome. In order to determine which prespecified candidate variables to include, we conducted variable selection using the random survival forest method in the entire sample while we were unaware of the trial-group assignments. We used imputation for missing values in an algorithm to select covariates for inclusion in a targeted minimum loss–based estimation model. A time-to-event analysis was based on the period from the time of transfusion until an outcome occurred. A two-sided test with a type I error of 0.05 was used to determine statistical significance.

Secondary Analysis Statistical analysis

The ratio of anti-S-RBD IgG antibody levels between unique CCP donors and D0 seronegative, unvaccinated recipients was calculated by dividing the geometric mean (GM) of donor AUC values by that of the CCP recipients.

We established correlates of protection using donor anti-S-RBD IgG levels by to methods—one based in virus neutralization and another using ROC analysis. For the first method, we established a functional cutoff value for binding antibody levels based in virus neutralization to delineate between high and low donor anti-S-RBD IgG AUC levels. Virus neutralization antibody at 1:40 dilutional titer has previously been reported as a correlate of protection in previous influenza studies [insert citation]. First, we calculated the upper limit of 95% confidence interval for the donor anti-S-RBD IgG AUC geometric mean at which the donor nAb is at 1:40 dilutional titer. We identified a GM of 2291 AUC with a lower limit of 1924 and upper limit of 2728 AUC. Recognizing that the antibody levels of seronegative CCP recipients were approximately 21.3

times lower than their respective donors, we further inferred the functional cutoff point for CCP recipients to also be 21.3 times lower than that of donors (128 AUC).

The RCDC curves were plotted[7] for control and CCP recipients anti-S-RBD post-transfusion. To calculate the antibody threshold level for early transfusion, a logistic regression model with hospitalization as the outcome and post-transfusion antibody level as the predictor was fitted for seronegative and unvaccinated participants who received early treatment. A ROC curve was plotted using the logistic regression model. An estimated optimal threshold value from the ROC curve that maximizes sensitivity and specificity was established by Youden's J statistics. The optimal antibody level associated with the estimated optimal threshold value from ROC was determined using the fitted logistic regression model. For late transfusions, the maximum percent hospital reduction on the two curves determined the antibody threshold level.

Spearman correlations were used to evaluate strength of association between titer and AUC units for antibody measurements. Predicted probabilities of hospitalization by the early versus late and high versus low categories were assessed using a Firth's logistic regression model due to complete separation in the dataset. Longitudinal seronegative recipient antibody data were first $\log_{10}$-transformed and analyzed using a linear mixed-effects regression model, adjusted for variant, age, sex, and BMI. An interaction term was included to examine how antibody levels changed over time by treatment (control or CCP) and hospitalization status. Predicted effects were graphed with 95% confidence intervals. Statistical analyses were performed using GraphPad Prism 8 (GraphPad Software) and Stata 17 (StataCorp).

Supplemental Figure 1 Conversion of anti-S-RBD AUC to ng/mL viral specific antibody. A) Determination of ng/mL of anti-S-RBD specific antibody levels in both post-transfusion recipients (n=33) and donors (n=55) with correlation to anti-S-RBD AUC. The ng/mL units were 1.8 fold greater than anti-S-RBD AUC comparing geomeans of the combined 88 samples tested. B) Using the strong correlation of determined ng/mL to S-RBD AUC, the 319 anti-S-RBDs AUC of unique donor units were converted to ng/mL (RBD AUCx1.8= ng/mL S-RBD antibody) and multiplied by 210 the average volume of transfusion to approximate a 1210 geomean for total mcg of viral specific S-RBD. C) Total full length spike ng/mL is approximately 4.3 times S-BD ng/mL (n=31) which translates to 5.2 total mg spike viral specific antibody dose per donor unit. D) Spearman correlation of donor anti-S-RBD IgG across different units of measurement (e.g., ng/mL, AUC, titer).

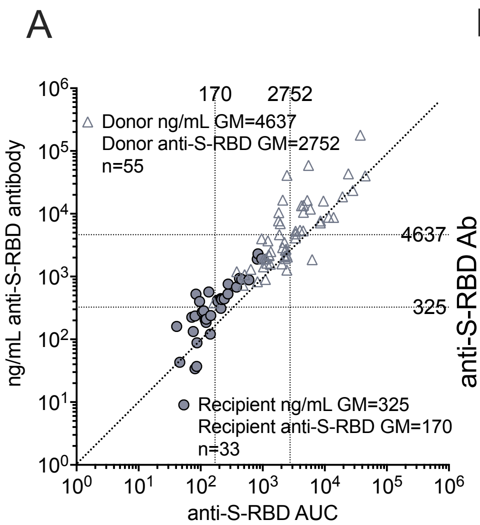

	S-RBD AUC vs. Donor ng/mL	S-RBD AUC vs. Recipient ng/mL
Pearson r		
r	0.6755	0.9533
95% confidence interval	0.4997 to 0.7978	0.9067 to 0.9769
R squared	0.4562	0.9087
P value		
P (two-tailed)	<0.0001	<0.0001

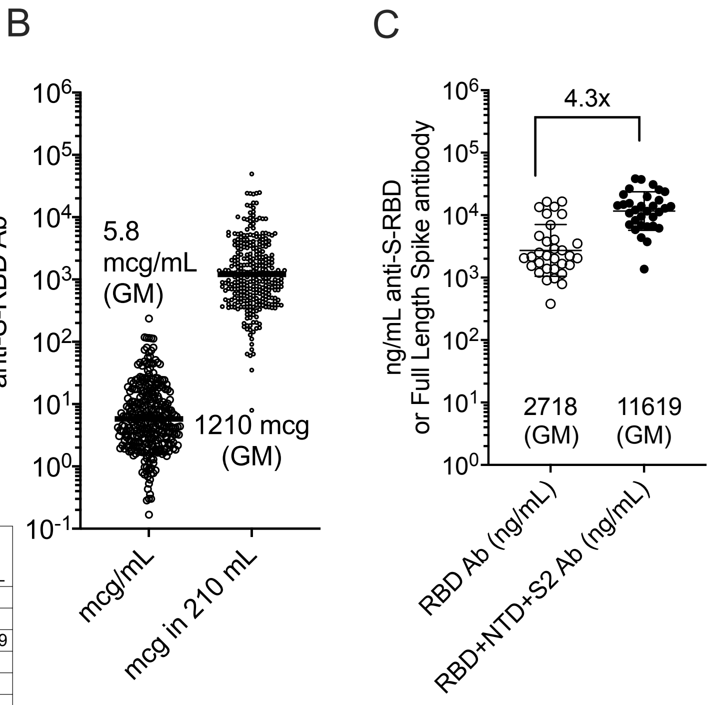

D

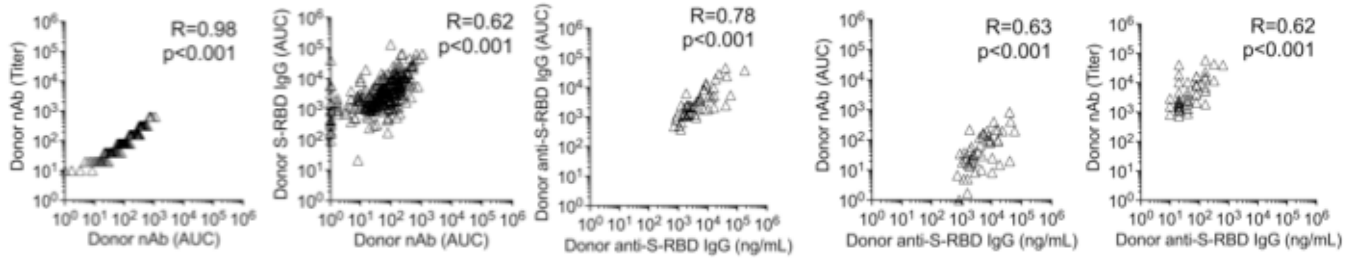

Supplemental Figure 2 Screen seropositive participants antibody levels stratified by transfusion days from symptom onset The 199 unvaccinated seropositive participant screen pretransfusion antibody levels stratified by days from symptom onset to transfusion. All point estimates are shown with error bars indicating the geometric mean with geometric SD. Numbers above the x-axis represent geometric mean (GM), the number in the group (n). The dashed line in B-E represents the upper post-transfusion 128 AUC recipient's threshold. All point estimates are shown with error bars indicating the mean with SD.

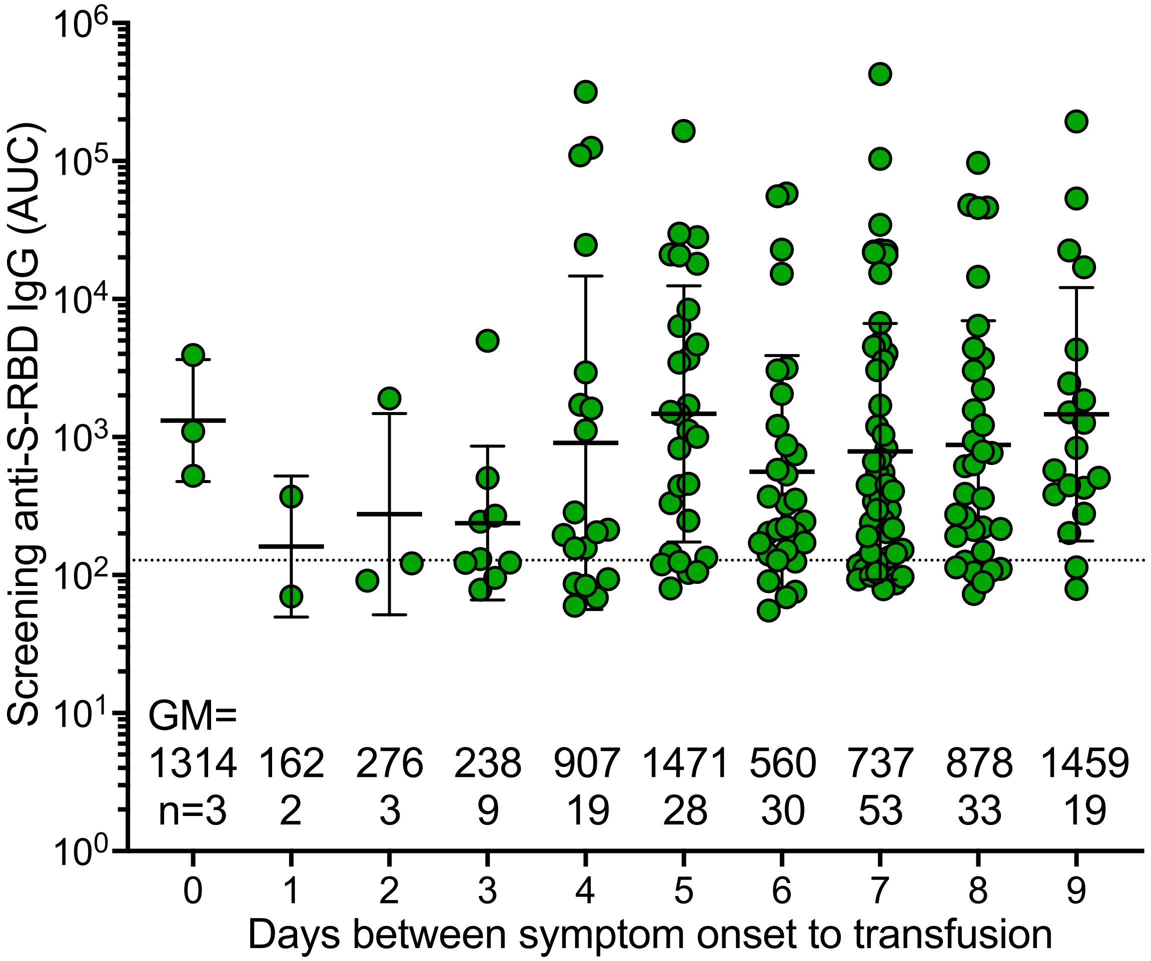

Supplemental Figure 3 Screen pre-transfusion nasal swab viral load determinations segregated by A) unvaccinated CCP or control plasma administration B)segregated by those hospitalized or not hospitalized C) Unvaccinated early or late control or CCP participants at screen D)Unvaccinated pre-Delta period participants were segregated into B) seronegative and seropositive populations by symptom duration in days to transfusion. Numbers above the x-axis represent geometric mean (GM), the number in the group (n), and percentage of PCR-positive samples (%) for each category. *** p<0.001, ** p=0.002 and * p=0.033 by non-parametric Kruskal-Wallis multiple comparisons test with Dunn's post-hoc corrections. All point estimates are shown with error bars indicating the GM with SD. The dashed lines indicate samples below the limit of detection of 330 viral copies.

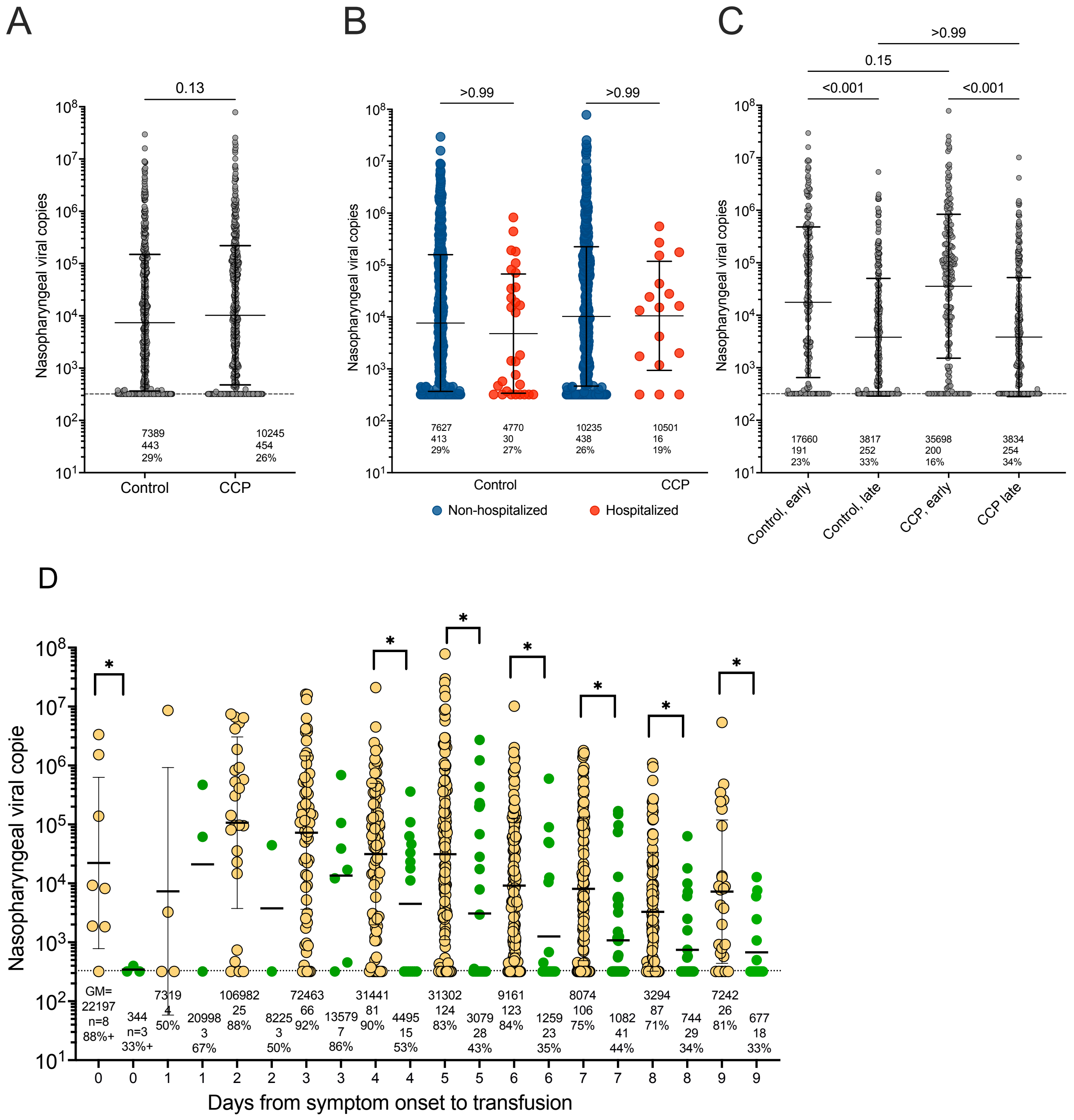

Supplemental Figure 4 Screen viral loads during the Delta period. During the Delta period there were only 77 (34%) participants unvaccinated to segregate into A) seronegative (n=42) and B) seropositive (n=35) groups by duration from symptom onset to transfusion. C) During the Delta period fully vaccinated participants (n=128) were antibody positive with an additional single recipient fully vaccinated, but seronegative with nasal viral load on day 0 of 320 (not graphed). All point estimates are shown with error bars indicating the GM with SD. Numbers above the x-axis represent geometric means (GM), the number in the group (n), and the percentage of samples PCR positive (%). The dashed lines indicate samples below the limit of detection of 330 viral copies.

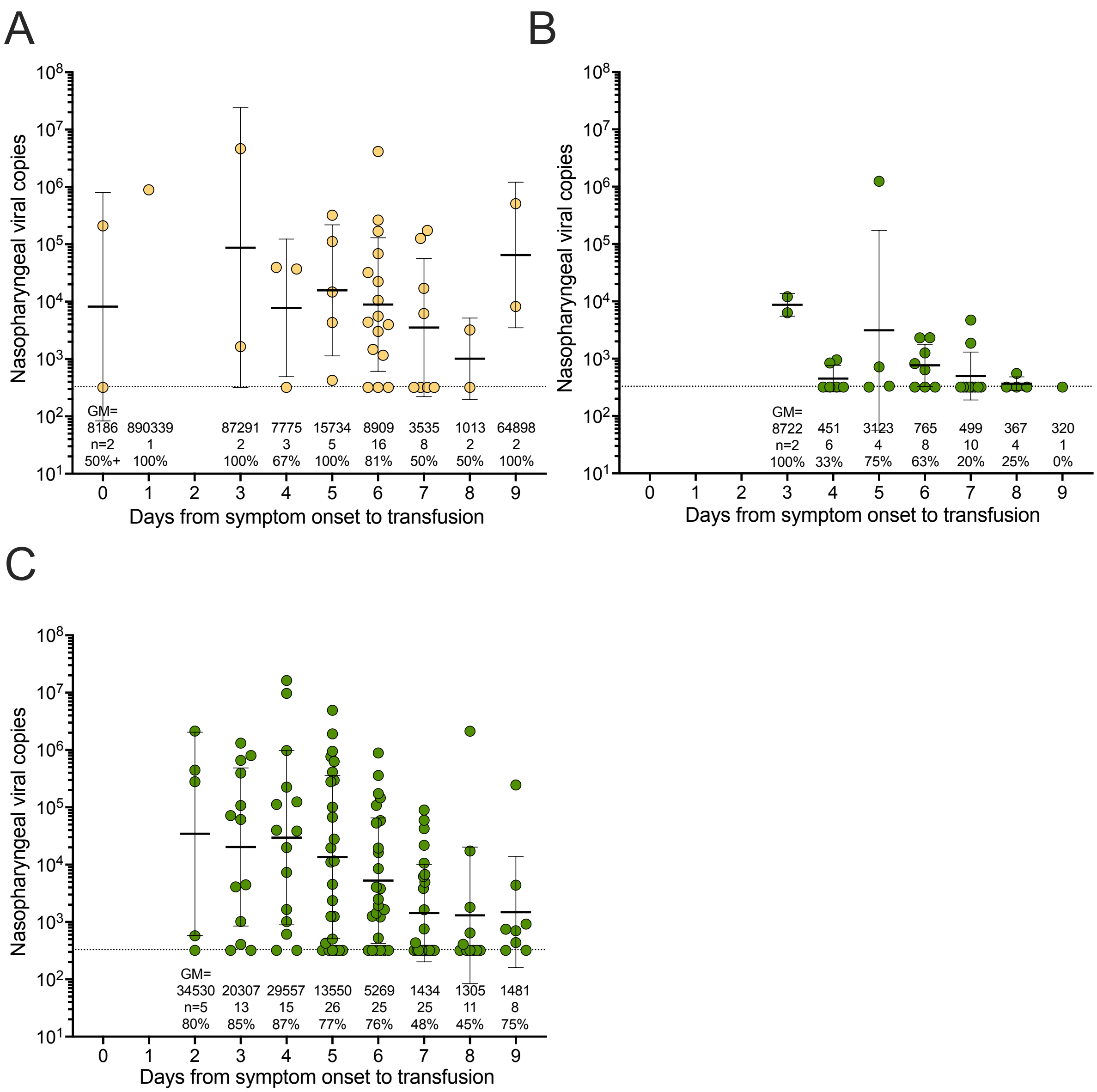

Supplemental Figure 5 Antibody levels three months post-transfusion. Unvaccinated recipients anti-S-RBD AUC antibody levels at Day 90 post-transfusion (excluding the 165 vaccinated during the follow-up visits) separated by A) CCP and control recipients B) both CCP and control recipients by SARS-CoV-2 variant period and vaccination status. Clear squares indicate donor, red squares indicate hospitalized recipients, and gray squares indicate both CCP and control non-hospitalized recipients. All point estimates are shown with error bars indicating the GM with SD. Numbers above the x-axis represent each category's geometric mean (GM) and number in the group (n). The dashed line in A, B represents the upper portion post-transfusion 150 AUC recipient's threshold, GM donor 3286 AUC and GM donor 6678 titer. *** p<0.001, by non-parametric Kruskal-Wallis multiple comparisons test with Dunn's post-hoc corrections.

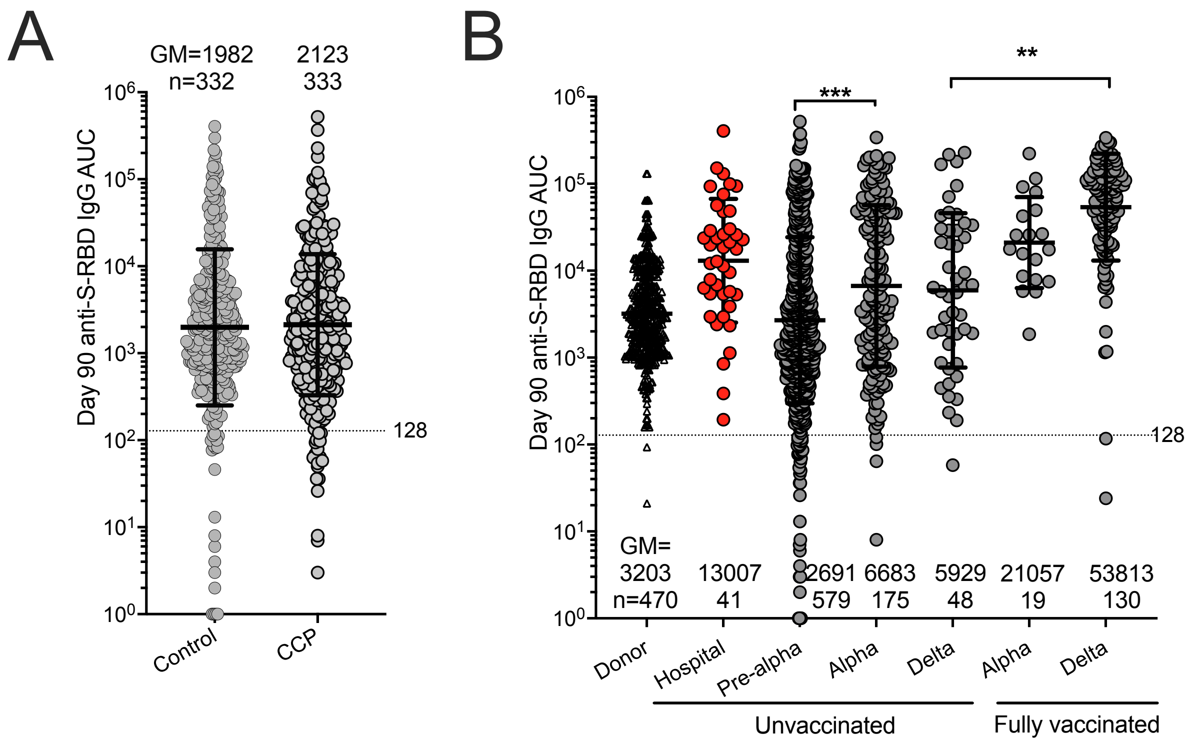

Supplemental Table 1
Data for ng/mL glucose based determination of viral specific antibodies

Donor anti-S-RBD AUC	Donor ng/mL	Donor anti-S-RBD AUC	Donor ng/mL	Recipient anti-S-RBD-AUC	Recipient ng/mL
163	381	3373	4781	41	160
381	1195	3374	4128	46	43
481	726	3386	5283	72	225
510	1071	3985	5472	76	133
642	1300	4211	13439	81	235
823	828	4491	5345	81	34
959	4008	4527	10541	84	524
1029	1429	4543	13633	86	37
1037	1893	5222	16344	87	88
1104	899	5456	58957	96	401
1132	1933	5850	11877	106	273
1167	1630	6242	1854	112	283
1214	3714	8485	7141	118	204
1251	2220	8508	7678	122	186
1268	2151	9489	15928	123	209
1329	1572	11553	8772	135	567
1350	2501	13866	8752	143	121
1791	7731	19253	18654	145	239
1816	10427	23969	43264	186	417
1858	4648	28291	23528	191	399
1918	6955	37345	178553	212	446
2035	1588	44727	40090	212	311
2091	2888			218	431
2097	16521			239	441
2229	3505			275	529
2288	2137			277	758
2313	2628			378	679
2321	1930			422	935
2375	2721			458	902
2439	2186			597	887
2446	1264			815	1895
2460	40860			839	2299
2498	2048			961	1922
**Geomean**		**2752**	**4637**	**170**	**325**

Supplemental Table 2 Firth's Logistic Regression Contrasts and P-values

Method of Cutoff	Quadrant Category	Predicted Probability	P-value
Virus Neutralization Antibody Cutoff	Early/Low	0.0636567	0.008
	Early/High	0.0086185	0.465
	Late/Low	0.0457482	0.045
	Late/High	0.0605447	0.007
ROC Cutoff	Early/Low	0.0673155	0.008
	Early/High	0.0082876	0.462
	Late/Low	0.0567985	0.002
	Late/High	0.0446232	0.174

Method of Cutoff	Quadrant Comparisons	P-value
Virus Neutralization Antibody Cutoff	Early/Low vs Early/High	0.145
	Late/Low vs Early/High	0.237
	Late/High vs Early/High	0.160
ROC Cutoff	Early/Low vs Early/High	0.124
	Late/Low vs Early/High	0.153
	Late/High vs Early/High	0.276

Supplemental Table 3 Fishers Exact table results for unvaccinated seronegative participants

Unvaccinated seronegative	hospital	no hospital	total	Hospital percent	p to all seroneg control	row p to early cont	row p to late control	p to all other CCP	p to early low CCP
all controls	31	238	268	12.0					
early control	20	147	167	12.0					
late control	11	191	201	5.0					
donor early high	1	87	88	1.1	**0.002**	**0.002**		0.13	0.21
donor early low	4	82	86	4.7	0.09	0.07			
donor late high	6	98	104	5.8	0.12		0.78		
donor late low	4	79	83	4.8	0.09		1		
donor early low and late	14	259	273	5.1					
recip nAb early high	0	85	85	0.0	**0.0002**	**0.0003**		**0.03**	0.058
recip nAb early low	5	81	86	5.8	0.21	0.18			
recip nAb late high	7	107	114	6.1	0.18		0.8		
recip nAb late low	3	73	76	3.9	0.08		1		
recip nAb early low and Late (high/low)	15	261	276	5.4					
recip rcdc early high	0	94	94	0.0	**0.0001**	**0.0001**		**0.01**	**0.017**
recip rcdcearly low	5	72	77	6.5	0.3	0.256			
recip rcdc late high	1	39	40	2.5	0.1		0.69		
recip rcdc late low	9	141	150	6.0	0.11		0.81		
recip rcdc early low and Late (high/low)	15	252	267	5.6					

Supplemental Table 4 ROC Analysis Threshold Values-Recipient anti-S-RBD IgG.
The AUC for the early recipient ROC analysis was 0.734, which was 100% sensitive, but not 100% specific (i.e., some people with lower levels were not hospitalized). The AUC for the late recipient ROC analysis was 0.533.

Efficacy Method	Early				Late			
	Placebo**	CCP			Placebo**	CCP		
		Total	High Ab	Low Ab		Total	High Ab	Low Ab
Overall (ignoring antibody threshold)								
Seronegative at screen								
N participants	162	171			200	190		
Hospitalized, n (%)	18 (11.1)	5 (2.9)			10 (5.0)	10 (5.3)		
Treatment Efficacy %		73.7				-5.3		
P-value*		0.004				1.0		
**Recipient anti-S-RBD IgG**								
Max Ab in hospitalized (excluding 1 late outlier)								
Threshold (AUC, log10)			>2.05	<=2.05			>2.58	<=2.58
N participants			94	77			40	150
Hospitalized, n (%)			0 (0)	5 (6.5)			1 (2.5)	9 (6.0)
Treatment Efficacy %			100.0	41.6			50.0	-20.0
P-value*			<0.001	0.35			0.70	0.81
ROC analysis								
Threshold (AUC, log10)			>2.06	<=2.06			>1.91	<=1.91
N participants			94	77			139	51
Hospitalized, n (%)			0 (0)	5 (6.5)			8 (5.8)	2 (3.9)
Treatment Efficacy %			100.0	41.6			-15.1	21.6
P-value*			<0.001	0.35			0.81	1.0

*Fisher's exact test; p-value comparing treatment group from its corresponding control group (early vs. late)
** excluding NA values (5 early, 1 late)

References

1. Sullivan DJ, Gebo KA, Shoham S, et al. Early Outpatient Treatment for Covid-19 with Convalescent Plasma. *N Engl J Med*. May 5 2022;386(18):1700-1711. doi:10.1056/NEJMoa2119657

2. Klein SL, Pekosz A, Park HS, et al. Sex, age, and hospitalization drive antibody responses in a COVID-19 convalescent plasma donor population. *J Clin Invest*. Nov 2 2020;130(11):6141-6150. doi:10.1172/JCI142004

3. Sullivan DJ, Gebo KA, Shoham S, et al. Early Outpatient Treatment for Covid-19 with Convalescent Plasma. *N Engl J Med*. Mar 30 2022;doi:10.1056/NEJMoa2119657

4. Lu X, Wang L, Sakthivel SK, et al. US CDC Real-Time Reverse Transcription PCR Panel for Detection of Severe Acute Respiratory Syndrome Coronavirus 2. *Emerg Infect Dis*. Aug 2020;26(8):1654-65. doi:10.3201/eid2608.201246

5. Li M, Beck EJ, Laeyendecker O, et al. Convalescent plasma with a high level of virus-specific antibody effectively neutralizes SARS-CoV-2 variants of concern. *Blood Adv*. 2022:2022.03.01.22271662. doi:10.1182/bloodadvances.2022007410

6. Park HS, Shapiro JR, Sitaras I, et al. Adaptive immune responses in vaccinated patients with symptomatic SARS-CoV-2 Alpha infection. *JCI Insight*. Mar 8 2022;7(5)doi:10.1172/jci.insight.155944

7. Reed GF, Meade BD, Steinhoff MC. The reverse cumulative distribution plot: a graphic method for exploratory analysis of antibody data. *Pediatrics*. Sep 1995;96(3 Pt 2):600-3.
